# #WhyWeTweetMH: Understanding Why People Use Twitter to Discuss Mental Health Problems

**DOI:** 10.2196/jmir.6173

**Published:** 2017-04-05

**Authors:** Natalie Berry, Fiona Lobban, Maksim Belousov, Richard Emsley, Goran Nenadic, Sandra Bucci

**Affiliations:** ^1^ Health eResearch Centre University of Manchester Manchester United Kingdom; ^2^ Division of Psychology and Mental Health School of Health Sciences, Faculty of Biology, Medicine and Health University of Manchester Manchester United Kingdom; ^3^ Spectrum Centre for Mental Health Research School of Health and Medicine Lancaster University Lancaster United Kingdom; ^4^ School of Computer Science University of Manchester Manchester United Kingdom; ^5^ Centre for Biostatistics Division of Population Health, Health Services Research & Primary Care, School of Health Sciences, Faculty of Biology, Medicine and Health University of Manchester Manchester United Kingdom; ^6^ Manchester Academic Health Science Centre Greater Manchester Mental Health NHS Foundation Trust Manchester United Kingdom

**Keywords:** mental health, Twitter, social media

## Abstract

**Background:**

Use of the social media website Twitter is highly prevalent and has led to a plethora of Web-based social and health-related data available for use by researchers. As such, researchers are increasingly using data from social media to retrieve and analyze mental health-related content. However, there is limited evidence regarding why people use this emerging platform to discuss mental health problems in the first place.

**Objectives:**

The aim of this study was to explore the reasons why individuals discuss mental health on the social media website Twitter. The study was the first of its kind to implement a study-specific hashtag for research; therefore, we also examined how feasible it was to circulate and analyze a study-specific hashtag for mental health research.

**Methods:**

Text mining methods using the Twitter Streaming Application Programming Interface (API) and Twitter Search API were used to collect and organize tweets from the hashtag #WhyWeTweetMH, circulated between September 2015 and November 2015. Tweets were analyzed thematically to understand the key reasons for discussing mental health using the Twitter platform.

**Results:**

Four overarching themes were derived from the 132 tweets collected: (1) sense of community; (2) raising awareness and combatting stigma; (3) safe space for expression; and (4) coping and empowerment. In addition, 11 associated subthemes were also identified.

**Conclusions:**

The themes derived from the content of the tweets highlight the perceived therapeutic benefits of Twitter through the provision of support and information and the potential for self-management strategies. The ability to use Twitter to combat stigma and raise awareness of mental health problems indicates the societal benefits that can be facilitated via the platform. The number of tweets and themes identified demonstrates the feasibility of implementing study-specific hashtags to explore research questions in the field of mental health and can be used as a basis for other health-related research.

## Introduction

### Background

Use of social media websites such as Facebook and Twitter is commonplace, with around 65% of American adults [1] and 66% of British adults [2] reporting ownership of at least one active social media account. High rates of social media use are also evident by individuals who experience mental health problems [3,4]. Research in the field of social media and mental health has largely focused on the potential harm of social media engagement. For example, researchers have observed or empirically evidenced associations between social media use and the occurrence and exacerbation of experiences associated with psychosis [5-7], mood disorders [8-10] personality disorders [10], eating disorders [11,12], and obsessive compulsive disorder [13]. However, others have reported that there are no associations between mental health problems and social media use and, in some cases, significant improvements in social functioning have been observed following social media engagement [14-16]. Mixed and correlational findings in the field and limitations in the methodological design of studies highlight the infancy of our understanding of the relationship between social media use and mental health [17-19]. In addition, much of the current research has focused on the use of Facebook, rather than Twitter, but the nature of the two sites and users differ extensively. For example, a recent comparative analysis of user behavior of individuals with Facebook and Twitter accounts demonstrated no significant overlap between Facebook “friends” and Twitter “followers,” and reported that Facebook was often used as the main platform for communication, whereas Twitter was used as a secondary platform [20]. In addition, user preference for the two different social media platforms has been found to differ based on user personality traits [21]. Therefore, caution needs to be taken when applying findings relating to Facebook to Twitter use.

Twitter (www.Twitter.com) is a popular microblogging site, with 313 million monthly users [22]. Twitter users are able to post 140-character limit posts or “tweets,” which others can respond to via retweeting, replying, or liking posts [23]. Such posts are often publicly accessible and, therefore, available for collection and analysis by researchers. As such, recent studies have collected tweets that included hashtags such as #depression, #schizophrenia, and #dearmentalhealthprofessionals to analyze mental health-related attitudes and experiences [24-26]. A recent editorial argued that the use of mental health-related hashtags facilitates connections, enables sharing without barriers, and provides the opportunity to voice opinions [27]. Furthermore, a mental health ambassador and educator with lived experience described the “helping hands” of Twitter that can guide people to safety [28]. However, there is little empirical research examining reasons why individuals use Twitter to discuss mental health problems.

### Aims of the Study

This study implemented the hashtag #WhyWeTweetMH to (1) examine why people use Twitter to discuss mental health problems and (2) investigate whether it is feasible for researchers to directly implement a Twitter hashtag that yields meaningful data for analysis. Twitter was specifically chosen as the social media platform of interest due to the prevalence and popularity of discourse surrounding mental health that is evident on the website.

## Methods

### Hashtag Development

**Figure 1:**
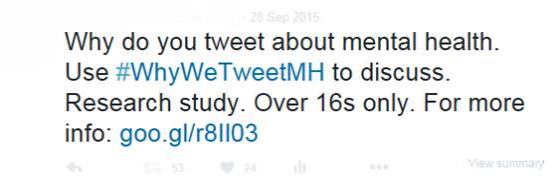
Example circulation tweet on the social media website Twitter using the #WhyWeTweetMH hashtag, research study disclaimer, and link for additional information.

Twitter allows users to post any information that they wish to share in the form of a 140-character tweet. Tweets posted by users can be “retweeted” so that any tweet an individual wishes to share can be posted on their Twitter profile for their followers to see. Twitter also affords users the opportunity to include hashtags within tweets, which can facilitate communication about, and efficient search for, a specific topic. To this end, the hashtag #WhyWeTweetMH was selected to be circulated on Twitter by the research team (see Figure 1). The decision to use the hashtag #WhyWeTweetMH was based on a number of discussions within the research team. The initial hashtag #WhyWeTweet was developed due to the small number of characters that would be used within response tweets and the alliterative and, therefore, memorable phrasing used. Additionally, popular mental health-related hashtags such as #MHawareness, #MHcare, and #MHservices use the acronym “MH” to refer mental health on the platform. Therefore, the letters MH were added during the development of the hashtag to ensure that the users were aware that the study was seeking reasons for discussing mental health specifically on Twitter.

### Data Collection

The first author (NB) posted the circulation tweet on the researcher’s own Twitter page; this was retweeted by other members of the research team. The researcher then individually contacted various mental health charities, campaigners, and advocates asking them to retweet information about the study. Initially, we were only seeking to collect responses from people with current or past experiences of mental health problems; however, some responses were written from other perspectives; for example, academics, clinicians, and charities. Therefore, it was decided that any tweet including the hashtag #WhyWeTweetMH would be analyzed. Frequent attempts were made by the research team to circulate the hashtag until no new tweets were posted including #WhyWeTweetMH. Collection of tweets using the hashtag occurred between September 2015 and November 2015.

Tweets were automatically collected and stored in a password-protected database. We used both the Twitter Streaming Application Programming Interface (API), for real-time data [29] collection, and the Search API for daily data collection [30] to minimize the risk of missing data due to any network connection failures. This approach ensured that if network errors resulted in a loss of real-time data, past data could still be obtained through the Search API. The hashtag #WhyWeTweetMH was used as the search and streaming keyword.

### Data Analysis

Once data collection was completed, all tweets including the hashtag #WhyWeTweetMH were imported to a password-protected Excel (Microsoft) file for qualitative thematic analysis. The Twitter handles (usernames) of users were removed to protect anonymity. During this process, retweets and any tweets posted to circulate the hashtag were removed. In addition, user geolocation was also collected and stored in a password-protected file.

Tweets containing #WhyWeTweetMH were visually inspected several times for common terms. Thematic analysis was used to identify the key reasons that users gave for discussing mental health problems on Twitter. The research team conducting the analysis consisted of a researcher with limited clinical experience and two clinical academics with extensive experience working with people with mental health problems. To ensure transparency and reliability, all tweets were read and analyzed by two members of the research team (NB and SB), who developed an emergent coding scheme to arrange the data. A hierarchical structure of descriptive headings and subheadings was produced and compared across all tweets. This structure was independently reviewed by FL and, as recommended by Turpin and colleagues [31], these categories were discussed again and refined with all members of the research team.

### Ethical Considerations

Ethical issues surrounding research using social media websites are complex and some individuals may perceive researchers “lurking” on Internet communities as intrusive [32]. However, as Twitter is considered a public platform, content posted on Twitter is publicly available to be used for research purposes [26]. Throughout the development and implementation of this study, several guidelines for Internet research were consulted and adhered to, specifically, the Association of Internet Researchers [33], the British Psychological Society [34], and INVOLVE [35]. In addition, ethical approval was granted by the University of Manchester Research Ethics Committee (ref: 15347). The use of these guidelines and consultation with the local ethics committee during the development process enabled the formulation of several methodological considerations to protect the safety and privacy of Twitter users.

As this was the first study of its kind to implement a mental health hashtag for research purposes, rather than collecting data from an already trending hashtag, new methods were employed to ensure that the study was ethically sound. First, the tweet circulating the hashtag #WhyWeTweetMH explicitly stated that the hashtag was being used for research purposes. The tweet circulating the hashtag also contained a link to an information sheet, which detailed a list of helplines that individuals would be able to contact should they require further support. The hashtag was also monitored several times a day to ensure that any potentially offensive or bullying comments to individuals who tweeted using the hashtag could be reported to Twitter. However, it is of note that none of the tweets identified contained offensive or bullying responses. Individual Twitter handles (usernames) were removed from the tweets to maintain confidentiality and, after thematic analysis, all tweets for presentation and publication purposes were paraphrased to ensure anonymity. Tweets were paraphrased by NB and reviewed by SB to confirm that the paraphrased tweets accurately reflected the content of the original tweets. Each paraphrased tweet was inputted into search engines and the Twitter search function to ensure that users’ profiles were not identified in the search results. In line with recommendations for the reporting of research conducted via Twitter [36], a full list of paraphrased Tweets is available as a Multimedia Appendix 1.

## Results

### Tweet Features

After the removal of retweets, a total of 132 original tweets posted by 90 different users contained #WhyWeTweetMH. The participant information sheet from the study was viewed 145 times during the study period. Respondents were located in the United Kingdom (n=44), the United States (n=22), Canada (n=4), South Africa (n=1), and Australia (n=1).The remaining users either listed a fictional location or did not have their location available (n=18). Respondents’ tweets were analyzed to determine whether experiences of using Twitter to discuss mental health problems were from personal or professional perspectives. The majority of the Twitter users who responded to the hashtag were identified from their responses as having personal experiences of mental health problems (n=50) and others were identified as working in the field of mental health (n=8). Inferences about user experience could not be made for the remaining respondents (n=32). We identified 4 themes and 11 associated subthemes. Some tweets presented several reasons for tweeting about mental health and are, therefore, applicable to multiple themes and subthemes. The frequency of themes and subthemes derived from the data, words used within subthemes, and the numbers of retweets and “likes” for each subtheme are presented in Table 1.

**Figure 2:**
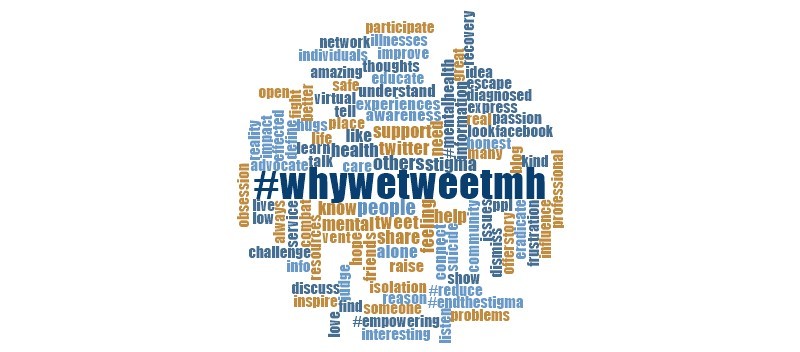
Word cloud reflecting the frequency of common words identified in tweets including the hashtag #WhyWeTweetMH.

Information regarding the frequency of common words in the tweets collected was recorded through splitting the text into single words. The words most frequently mentioned in the tweets were (1) stigma; (2) support or supporting; (3) alone; (4) connect; (5) awareness; (6) others; and (7) share or sharing appear in Figure 2, which was created using QSR International’s NVivo 11 software. These terms reflect some of the key themes and subthemes resulting from the tweets.

**Table 1:** Themes and subthemes derived from the #WhyWeTweetMHhashtag and the associated frequencies of retweets, “likes,” and popular words for each of the subthemes.

Theme and subthemes	Tweet frequency	Number of retweets	Proportion of tweets retweeted n (%)	Number of “likes”	Proportion of tweets “liked” n (%)	Commonly used words within subthemes	Word frequency
**Sense of community**							
To connect or socialize and reduce isolation	47	55	25 (53)	61	27 (57)	Alone Connect Others People or ppl Friends Isolation Community	13 11 9 8 4 4 4
To send and receive messages of hope and support	35	44	20 (57)	49	18 (51)	Support or supporting Hope Help or helps Hugs	14 5 4 3
To share and receive information	21	20	9 (43)	15	9 (43)	Share or sharing Information or info Resources Learn	9 7 5 5
**Stigma and awareness**							
To combat stigma	23	40	15 (65)	29	14 (61)	Stigma Combat Eradicate	19 3 2
To raise awareness	22	42	13 (59)	27	13 (59)	Awareness Raise Educate Understanding	11 7 4 3
To fight and campaign	11	29	9 (82)	15	7 (64)	Services Advocate Improve	3 3 3
**Safe space for expression**							
To share honest experiences without feeling judged	32	23	15 (47)	44	16 (50)	Experiences Share or sharing Honest Judge Safe	8 8 5 4 3
To vent, give people a voice, and feel heard	21	25	12 (57)	29	9 (43)	Vent Express Frustration	6 3 2
Perceived benefit over Twitter and other social media platforms	7	6	4 (57)	17	5 (71)	Facebook Networking or media	2 2
**Coping and empowerment**							
To escape	4	0	0 (0)	2	2 (50)	Escape Distract Suspend	2 1 1
Empowering form of self-monitoring and management	11	7	4 (36)	10	7 (64)	Empower Resilience Monitor Manage	1 1 1 1

### Theme 1: Tweeting About Mental Health Provides a Sense of Community

The overall sense of a “Twitter community” was evident through the explicit use of the word “community” in some of the tweets. The terms “Twitter friends” and “virtual hugs” were also prevalent, which implies a reciprocal relationship within the Twitter mental health community. In total, 51% (42/83) of the tweets included within this theme were retweeted and 53% (44/83) received “likes” from other users.

#### Tweeting to Connect, Socialize, and Reduce Isolation

Some users expressed that Twitter is the only setting where they are able to connect and socialize with others. The use of Twitter for some people as the sole avenue for communication may be due to the accessible nature of websites:

Because it is...the one space I can speak with people.

Because I am with friends even when I am unable to go out.

Users also commented that tweeting about mental health provided them with their only opportunity to connect with others with shared understanding:

I am able to communicate with other people with the same experiences...

Additionally, tweeting about mental health was perceived by users as a way to reduce feelings of isolation and loneliness and allowed them to show others and themselves that they are not alone:

...so I do not feel that I am the only person with MH concerns.

If it helps even one person recognize they aren’t alone in their pain.

#### Tweeting to Send and Receive Messages of Support and Hope

Support was detailed in many tweets, with users expressing that they often tweet about mental health to provide and receive messages of hope and support:

I enjoy supporting people and receiving support from them.

Suicide might be complicated but extending a hand to someone is simple and it may save their life...

I tweet humour to show people that there is light at the end of the dark tunnel...

Some users also expressed that by sharing their experiences on Twitter, they could help people who were facing similar challenges:

So that, perhaps, my tweets and experiences may help others. Even if it’s only one person.

Twitter was perceived as an accessible avenue for support due to the instantaneous nature of the responses:

I am able to get fast, insightful and appreciated support in a way that’s meaningful for me...

#### Tweeting to Share and Receive Information

Some users also reported that tweeting about mental health provided them with the opportunity to ask questions, learn more about mental health, and to seek and signpost useful resources:

To advise, support, and to ask questions...

Tweeting about mental health helps people to obtain helpful info they would not normally hear about.

### Theme 2: Tweeting About Mental Health to Combat Stigma and Raise Awareness

Tweets that contained information about using Twitter to raise awareness of mental health problems, combat stigma, and fight and campaign received the largest proportion of retweets (65%, 31/48) and “likes” (58%, 28/48). Additionally, 82% (9/11) of the tweets in the subtheme tweeting to fight and campaign received retweets and 64% (7/11) were “liked.” The high proportion of responses to such tweets may be due to other users sharing these tweets in an attempt to further campaign for people experiencing mental health problems and the high number of followers that campaigners or advocates may have on the site. Tweeting to raise awareness, combat stigma, and fight and campaign were often detailed by users as an attempt to achieve a final outcome; for example, developing empathy and compassion, to show people that others care and to provide hope for the future:

To raise awareness, stop stigma, create networks, & build empathy & compassion is #WhyWeTweetMH.

To bring buried, misjudged, and shameful disorders out of the darkness. To relieve the struggle of those still to come.

#### Tweeting to Combat Stigma

Many of the antistigma tweets contained particularly strong and emotive language such as “combat,” “demolish,” and “fight” to describe the concept of using Twitter to address stigmatizing attitudes. In addition, some Twitter users embedded the already popular hashtag #endthestigma into their #WhyWeTweetMH responses:

We do not only need to challenge stigma we have to eliminate stigma...

...to attempt to battle stigma...

#### Tweeting to Raise Awareness

Some users reported that Twitter was a common starting point for important conversations about mental health problems:

...begin speaking about what’s actually important...

To begin the conversation and open the barriers...

Additionally, some tweets also included already trending mental health awareness hashtags; for example, #mentalhealthawareness and #everyonesbusiness.

#### Tweeting to Fight and Campaign

Some people saw Twitter as an avenue for campaigning about mental health, which allowed them to represent others experiencing mental health problems:

To inform, empower, and inspire. We must advocate for and show others how to advocate for themselves.

...An advocate told me that my voice was required on here to confront the “Master Narratives” about mental health, trauma, and suicide.

### Theme 3: Tweeting About Mental Health Because Twitter Is a Safe Space for Expression

Twitter was perceived as a safe setting in which users could discuss mental health honestly and openly without feeling judged by others. Perceptions of safety in comparison to other social media platforms were also evident in some tweets. On average, just under half of the tweets assigned to this theme were retweeted (48%, 22/46) and over half were “liked” by other users (54%, 25/46).

#### Tweeting to Share Honest Experiences Without Feeling Judged

Several respondents noted that the perceived anonymity of Twitter allowed them to feel safe and, therefore, felt comfortable in being open and honest about their experiences of mental health problems:

I tweet because I am able to be anonymous so honest...

Some users also stated that Twitter allowed them to share thoughts and feelings relating to mental health on Twitter without feeling judged by others. Although efforts have been made to reduce stigma and judgmental attitudes toward mental health problems, these issues are still prevalent in society [37,38]. However, perceptions of safety and accepting attitudes reported by users suggest that Twitter may provide a protective platform for communication and expression that is, perhaps, not available in everyday life:

...because I’m never dismissed by my Twitter friends as being over sensitive, needing attention, or not making enough of an effort.

#### Tweeting to Vent, Have a Voice and Feel Heard

Users expressed that tweeting about mental health was a release and provided them with an outlet to voice any worries or concerns they were experiencing:

...When I tweet about mental health it’s a release...I also want the world to see how rubbish I feel...

Respondents also reported that they tweeted to share their experiences of the mental health system and service availability:

I can voice my infuriation with the professional support systems or lack of.

I like to tweet when I am angry at mental health services, so that even if nothing is resolved, my complaint is still public.

Twitter was perceived to be a platform on which to vent because some users felt unable to share thoughts and feelings in face-to-face settings with people who they personally knew.

Mainly I use Twitter as a soapbox so I am able to avoid burdening my friends...

#### Benefit of Twitter Over Other Social Media Websites

Some users reported that discussing mental health on Twitter was more appropriate than other platforms because they did not feel judged by others on Twitter and could avoid the heavily embellished version of peoples’ lives evident on Facebook:

I tweet about mental health problems, information, and feelings because no one judges me on Twitter, unlike other social networking sites...

Facebook is the sparkly sunny version of people, Twitter is the authentic version...

### Theme 4: Tweeting About Mental Health Is an Empowering Coping Mechanism

A smaller number of users revealed tweeting about mental health as a self-directed coping mechanism, which enabled them to escape from challenges faced in daily life; recognize and reflect on thoughts, feelings, and experiences; and facilitate feelings of empowerment. There were comparatively far fewer retweets of responses included in this theme than the other themes noted (27%, 4/15), although the proportion of “liked” tweets was similar (60%, 9/15).

#### Tweeting About Mental Health Provides an Opportunity to Escape

Some users reported that using Twitter provides them with the opportunity to escape from the “real-world” and distract themselves from difficult thoughts or feelings:

To distract myself from my mental health. I enjoy being able to laugh and joke on Twitter—that’s the part of me that I like...

...interrupt my irrational and obsessive thoughts—it does work.

#### Tweeting About Mental Health as an Empowering Self-Management Strategy

The concept of using Twitter as a mood monitor was evident in several tweets, as it allowed respondents to express themselves on Twitter and reflect back on the tweets to recognize their thoughts and feelings over time:

I began tweeting so that I will someday be able to look back at how bad things have been, as blogging was too much for me...

My Twitter timeline performs as a sort of mood monitor for myself and those who personally know me...

The potential strength of Twitter as a coping mechanism was evident in some tweets, which stated that Twitter was “cheaper than therapy,” “Twitter saves lives,” and the inclusion of the hashtag #lifehack.

Some users also commented that they felt empowered by tweeting about their mental health, which suggests that tweeting about mental health can be an empowering experience:

Tweeting’s empowering...

## Discussion

### Principal Findings

The aims of this study were to (1) explore reasons why people use Twitter to discuss mental health problems and (2) examine whether study-specific Twitter hashtags can be implemented by researchers as a method for data collection. The collective experiences noted are indicative of the positive role that Twitter can provide in mental health discussions and the number of tweets collected suggests that the circulation of study-specific hashtags on Twitter is a feasible avenue for investigating mental health-related phenomena.

The content expressed in collected tweets conveyed the notion of a “Twitter community” that allowed communication to flourish, awareness to be raised, stigma to be fought, and support that could be both offered and received. These perceived functions of Twitter support previous assertions that the platform provides a space for mental health-related discussions [26,27] and self-disclosures [39] and the wider literature regarding the social ties, sense of community, and support mechanisms that can be developed when communicating about health and experiences on the Internet [40-42]. Sense of belongingness and integration within a community can benefit an individual’s mental health and may be a protective factor in the development and exacerbation of symptoms associated with mental health problems [43-45]. In addition, social disconnectedness is often associated with higher rates of relapse [46], increased mortality [47], and poorer physical and mental health [48]. Therefore, being a member of a large Twitter mental health community may act as a protective factor by facilitating communication and support. Furthermore, combatting stigma and raising awareness were key reasons identified for tweeting about mental health, which may help foster the sense of community that was evident in the tweets.

The positive evaluations of the Twitter mental health community for the provision of support may, in part, be due to the value of a shared understanding on Twitter. Some users noted that Twitter allows them to communicate and receive support from others with similar experiences. There has been a growing movement in psychological practice toward the inclusion of peer support approaches, whereby individuals with experience of mental health problems provide support for people with similar experiences [49]. The potential value of peer support has been widely discussed in the literature and is associated with improved functioning, empowerment, and confidence [49], reductions in hospital admissions [50], and increased social networks and wellbeing [51]. The notion that social media could provide an accessible avenue for peer support is not new necessarily. A recent commentary regarding social media usage in severe mental health problems reported that social media could facilitate help-seeking behaviors, reciprocal support, and antistigma campaigns [52]. In addition, Naslund and colleagues [53] analyzed comments on videos created by individuals experiencing severe mental health problems on the video sharing platform YouTube (www.youtube.com). The authors reported that there was evidence of naturally occurring peer support within the comments, which provided supportive messages and coping strategies and reduced isolation. As such, the findings from this study support the view that social media websites, in this case, Twitter, could be a valuable tool for people who experience mental health problems.

Many users also noted that they were able to access resources and information on Twitter that they ordinarily would not be able to retrieve. The availability and subsequent use of Web-based material may help to facilitate self-directed psychoeducation, which is a potentially effective psychological intervention [54]. Therefore, resources on Twitter could be employed by users as a self-directed psychoeducation intervention. In addition, access to Web-based health-related information is reportedly beneficial for improving health behaviors, awareness and care of conditions, and could facilitate help-seeking [55-57]. Individuals experiencing mental health problems, clinicians, and academics could also use Web-based resources shared by other Twitter users to remain informed about recent advances in clinical practice and current research in the field. Some of the tweets that included #WhyWeTweetMH also contained other trending hashtags; for example, #everyonesbusiness and #mentalhealthawareness. The inclusion of such hashtags illustrates the popularity of incorporating mental health hashtags within tweets and supports the notion that hashtags can be an effective method to facilitate communication about specific topics.

The use of Twitter to share experiences of mental health services was also evident in some of the tweets and supports previous conclusions that mental health services could use Twitter to receive feedback on the care that they provide [26]. Users also reported that Twitter allowed them to be open and honest about their experiences. Providing the individual consents, mental health professionals may have the opportunity to review clinically relevant information disclosed by users on Twitter accounts that they may ordinarily feel uncomfortable sharing in a formal clinical setting. However, further research assessing Twitter user and health care professional views toward the collection of clinically relevant information via Twitter is warranted. Additionally, the use of Twitter as a coping mechanism, which is evident in some tweets, suggests that social networking tools may be popular as a component for psychological interventions.

### Feasibility of Circulating a Study-Specific Hashtag on Twitter for Research Purposes

Previous research using Twitter hashtags for data collection has relied on the analysis of already trending hashtags [24-26]. Therefore, the second aim of this study was to assess the feasibility of circulating a study-specific hashtag for research purposes. As there was no precedence for what constituted a “sufficient” number of tweets for research purposes, we gathered a sufficient number for qualitative analysis, demonstrating that it is feasible to employ research hashtags on Twitter. Indeed, the ethical integrity of providing a study-specific hashtag that users are aware is being used for research, rather than collecting preexisting data, may negate the potential disadvantages of reduced data. However, when considering the implementation of study-specific hashtags in mental health research, researchers should remain mindful about the ethical considerations associated with asking people to tweet about their mental health and moral issues surrounding the duty of care toward users who choose to share their views. Additionally, further research should seek to identify Twitter users’ views about the collection of their data using Twitter hashtags to determine whether or not they find this approach acceptable or potentially intrusive. Researchers seeking to use study-specific hashtags in future work may also wish to consider creating a specific Twitter account for research studies. The circulation of a research hashtag on a study-specific Twitter account may allow users to feel more comfortable tweeting about their experiences due to the anonymous nature of a study account; therefore, potentially increasing responses.

### Study Strengths

There were some strengths and limitations to the novel methodology employed. First, the ethical integrity of the study was a considerable strength. Specifically, a research disclaimer was included in the tweets circulating #WhyWeTweetMH, usernames were removed from all tweets before analysis, and tweets were paraphrased after analysis for presentation and publication purposes. Additionally, the investigation of tweeting behavior took place in the setting in which the behavior directly occurred, which ensured that respondents to the hashtag were active Twitter users. The use of both the streaming API and search API to collect tweets reduced the likelihood of missing data. The truly interdisciplinary nature of the research team (ie, computer scientists and psychologists) ensured that the approach to collect tweets was technologically and methodologically sound, and the research question, analysis of tweets, and implications for clinical practice were appropriate. Importantly, conducting this research on Twitter allowed people to provide views for a study without the constraints of traditional research such as location, time pressures, and effort required.

### Study Limitations

Some users may have chosen not to tweet using #WhyWeTweetMH due to the disclaimer that data would be used for research purposes, which may have led to missing data. Additionally, the use of Twitter to obtain reasons for tweeting about mental health may have led to biased responses of positive experiences, as people who do not use Twitter due to negative experiences will not have been able to detail reasons for not tweeting. As such, future research should seek to explore both the potential positive and negative experiences people have encountered when using Twitter to discuss mental health. The publicly accessible nature of Twitter may have also resulted in some users observing others responses and adapting their reasons for tweeting accordingly. The study also relied on the assumption that respondents actively tweeted about mental health and the 140-character limit of tweets may have prevented users from being able to give an in-depth insight about their reasons for tweeting. Therefore, the amount of material available for an in-depth exploration of tweet content was limited and could be utilized further as a method to identify a broad sample and purposively select participants from this sample for further questioning. It is generally seen as good practice to ask participants whether they agree that the analyzed data and paraphrased quotes accurately captured discussions during qualitative interviews. However, this is not feasible via Twitter due to the ethical issues surrounding directly contacting individuals tweeting with the hashtag. Additionally, tweets were limited to English-speakers, which may impact on the generalizability of the findings. To prevent the collection, analysis, and dissemination of potentially identifiable information, only user location (city and country) and the content of tweets containing #WhyWeTweetMH were collected. Although the majority of respondents indicated in the tweets that they experienced mental health problems, for some, interpretations regarding personal or professional experience could not be made. Finally, information such as diagnosis, age, and gender could not be collected due to the ethical considerations surrounding the collection of identifiable information without specific user consent.

### Conclusions

The number of tweets collected in the study and the thematic analysis applied demonstrates the feasibility of researchers directly implementing a hashtag for mental health research. Furthermore, the unique methodology employed resulted in the development and identification of several ethical considerations to ensure the safety and anonymity of Twitter users. The findings from #WhyWeTweetMH tweets suggest that individuals may actively use Twitter to discuss mental health as way of developing a sense of belonging within a community, accessing support, challenging stigma and raising awareness, sharing experiences, and as an empowering coping mechanism. Future research is planned to explore whether Twitter users are open to their data being used for research purposes and the acceptability of using Twitter as an avenue for evidence-based psychological interventions. In addition, further work regarding clinician views about Twitter use in mental health and how Twitter could help or hinder clinical practice should be considered. Although potential drawbacks of Twitter use must be considered, for example, cyberbullying and Web-based predators, the strong expressions within the tweets suggest that respondents to #WhyWeTweetMH have experienced Twitter as welcoming and supportive and a useful forum for an open and honest dialog about mental health.

## Appendix

Multimedia Appendix 1 Paraphrased versions of tweets that included the #WhyWeTweetMH hashtag.

## Abbreviations

API: Application Programming Interface
