# Supplementary material for: #WhyWeTweetMH: Understanding Why People Use Twitter to Discuss Mental Health Problems
Source: J Med Internet Res. 2017 Apr 5;19(4):e107. doi: 10.2196/jmir.6173 (PMC5399219; doi:10.2196/jmir.6173)
Supplement: Multimedia Appendix 1 [file jmir_v19i4e107_app1.pdf]

Appendix

Table S1. Paraphrased versions of tweets that included the #WhyWeTweetMH hashtag.

User	User Location	Experience	Paraphrased Tweets	Re-tweet frequency	'like' frequency
1	United Kingdom	Personal experience	I use Twitter as a place to vent, let go of worries & share with similar people. It provides a feeling of a community, which is great #WhyWeTweetMH	2	4
			I like to tweet when I am angry at mental health services, so that even if nothing is resolved, my complaint is still public. #WhyWeTweetMH	3	5
			Mainly I use Twitter as a soapbox so I am able to avoid burdening my friends #WhyWeTweetMH	1	2
2	United Kingdom	Personal experience	Twitter helps me realise that I am not alone with my diagnoses #WhyWeTweetMH	0	0
3	United Kingdom	Unknown	Nobody's alone!	1	0
4	United Kingdom	Personal experience	On Twitter I don't have to conceal or censor how I am feeling. It's okay to not be all right #WhyWeTweetMH	3	4
5	United Kingdom	Personal experience	I tweet about mental health problems, information and feelings because no one judges me on Twitter, unlike other social networking sites #WhyWeTweetMH	2	6
6	Unknown	Personal experience	I tweet because I am able to be anonymous so honest. I am able to communicate with other people with the same experiences, remain informed &inform other people #WhyWeTweetMH	1	1
7	United Kingdom	Personal experience	I tweet for precisely the same reasons as any other person. I am not defined by my mental health #WhyWeTweetMH	1	3
8	United Kingdom	Personal experience	I tweet because, like the majority of people, I enjoy being able to connect and socialise. #WhyWeTweetMH	1	1
			I enjoy supporting people and receiving support from them. #WhyWeTweetMH	2	3
			I also tweet so that I am able to say to people how adorable their pets are & obtain more pictures of pets to satisfy my obsession with pets! #WhyWeTweetMH	0	0
9	Unknown	Personal experience	#WhyWeTweetMH because it is the one space I feel safe to & it is the one space I can speak with people	2	4
10	United Kingdom	Personal experience	I tweet because I have spent way too long staying quiet about my battles and successes. #WhyWeTweetMH	1	2
11	United Kingdom	Personal experience	Because on Twitter I do not have to see the "successful" personal narratives of other people. #WhyWeTweetMH	1	1
12	Canada	Unknown	I tweet to terminate stigmatising views. #WhyWeTweetMH	1	1
13	United Kingdom	Personal experience	I am able to get fast, insightful and appreciated support in a way that's meaningful for me. I'm especially fond of virtual	2	4

			hugs #WhyWeTweetMH		
			My Twitter timeline performs as a sort of mood monitor for myself and those who personally know me #WhyWeTweetMH	1	1
			It's a space I can go to when I am unable or reluctant to go anywhere else #WhyWeTweetMH	1	1
			I can voice my infuriation with the professional support systems or lack of #WhyWeTweetMH	1	0
			Face facts, there are some incredible people on Twitter whose strength is something to admire #WhyWeTweetMH	2	7
14	United Kingdom	Personal experience	To distract myself from my mental health. I enjoy being able to laugh and joke on Twitter - that's the part of me that I like #WhyWeTweetMH	0	0
			#WhyWeTweetMH…I am really chatty, enjoy speaking with people with the same interests.	0	1
			#WhyWeTweetMH When I tweet about mental health it's a release… I also want the world to see how rubblish I feel…but I use Twitter to support me & try & forget	0	1
			#WhyWeTweetMH interrupt my irrational obsessive thoughts - it does work.	0	1
15	United Kingdom	Unknown	The use of social media when experiencing MH problems can help people escape	0	1
16	United Kingdom	Personal experience	Facebook is the sparkly sunny version of people, Twitter is the authentic version #WhyWeTweetMH	1	7
			I began tweeting so that I will someday be able to look back at how bad things have been, as blogging was too much for me #WhyWeTweetMH	0	1
			#WhyWeTweetMH - I can see that I can share my reality and there will be people who understand who will support me through the most difficult times	0	0
			#WhyWeTweetMH so I am able to check in with others around tea	0	1
			#WhyWeTweetMH it is a safe place to vent & to have people assess whether your thoughts about an incident were right or wrong	0	0
17	United Kingdom	Unknown	Because why should I not? Because people are able to tweet about whatever so why not something significant? #WhyWeTweetMH	2	2
18	Unknown	Personal experience	#WhyWeTweetMH. A space to express, vent, share experiences, learn, to not feel isolated, take part in wider peace	1	0
19	Unknown	Personal experience	Need, awareness, understanding, outreach, exasperation, loneliness. #WhyWeTweetMH	1	0
			Six months since a couple of decent people committed suicide, in those six months, basically no checkins from people #WhyWeTweetMH	0	0
			#toryconference Mental health only related to criminality? Mental health problems only related to poor education?	0	0

			#WhyWeTweetMH		
			In spite of my erudition, dealing with blackouts, intense GAD, depression & dissociation, on my own, Trying to learn & retain. #WhyWeTweetMH	0	0
20	United States	Personal experience	#WhyWeTweetMH for support, so I do not feel that I am the only person with MH concerns	3	7
			#WhyWeTweetMH so others can know that they are not alone. To take away the stigma about getting help for mental health	1	2
21	United States	Personal experience	To share, to find out about resources. To be heard. To realise 'you are not alone' #WhyWeTweetMH Number One: To release and let it be.	0	0
			…sometimes venting on Twitter is better than any other space. #lifehack #WhyWeTweetMH	0	0
			Tweeting’s empowering #WhyWeTweetMH	0	0
22	United Kingdom	Personal experience	#WhyWeTweetMH because it feels safe to reveal honest thoughts and emotions on here. People choose to follow you, not because they are your ‘friend’	0	0
23	United States	Personal experience	#WhyWeTweetMH I am able to connect with friends, acquaintances & other people even when I can’t get out of the house.	5	3
24	Unknown	Personal experience	There’s no one who can appreciate my mental health problems like other Trans people. I am connected to lots of Trans People via Twitter. #WhyWeTweetMH	0	4
25	United Kingdom	Personal experience	#WhyWeTweetMH - honest self-expression, seek & receive support, begin a dialogue, represent ourselves	1	0
26	United States	Personal experience	#WhyWeTweetMH because I’m never dismissed by my Twitter friends as being over sensitive, needing attention or not making enough of an effort	2	2
			#WhyWeTweetMH because my friends on Twitter will support me at all times & won't say that they do not care, or they won't 'enable' me by listening	0	0
27	United States	Personal experience	Connecting with other people with similar experiences and feelings makes me feel less alone, more certain I am able to survive #WhyWeTweetMH	1	0
28	United Kingdom	Personal experience	Because when I am feeling down, my Twitter friends never tell me to get over it. #WhyWeTweetMH	0	0
			So I can see that I am not alone. #WhyWeTweetMH	0	0
			So that, perhaps, my tweets and experiences may help others. Even if it's only one person. #WhyWeTweetMH	0	0
			Because Twitter is the only place where I feel I can honestly talk about my mental health without being judged. #WhyWeTweetMH	0	0
			Because tweeting about my thoughts and feelings helps me to make sense of them, when nobody in real life will listen. #WhyWeTweetMH	0	0
			Because I am with friends even when I am unable to go out.	0	0

			#WhyWeTweetMH		
			Because my Twitter friends extend support, guidance and virtual hugs if I need them instead of dismissing me as being moody and over emotional #WhyWeTweetMH	2	6
29	Canada	Personal experience	#WhyWeTweetMH it prevents isolation, it is cheaper than therapy, followers carry out genuine acts of kindness, I can relax my sleepy bones whilst doing it	0	2
30	Unknown	Personal experience	to vent & dodge the drama of Facebook. #WhyWeTweetMH	0	0
31	United States	Personal experience	vent & to deal with and make sense of my thoughts. #WhyWeTweetMH	0	0
32	Canada	Personal experience	Because on here people get me and do not make judgments about me. I do not have to explain things and the support is incredible! #WhyWeTweetMH	0	1
33	United States	Unknown	I use Twitter to help and educate others about mental health plus more things #WhyWeTweetMH	0	0
34	United Kingdom	Personal experience	To begin the conversation and open the barriers. To offer support to the effected & the affected. #WhyWeTweetMH	0	0
			Tweeting for me eradicates the sense of isolation. Being honest can create networks & draw on forgotten strength #WhyWeTweetMH	1	1
			I tweet for virtual hugs #WhyWeTweetMH	1	0
			The inspirational and encouraging tweets should continue every day - not only for this weekend. They have definitely provided me with strength #WhyWeTweetMH	2	0
35	United Kingdom	Personal experience	To advise, support & ask questions…every so often to say that I feel rubbish today as well! #WhyWeTweetMH	0	0
36	United Kingdom	Professional experience	#WhyWeTweetMH - to increase awareness that, like physical health, help should be received for mental health	0	0
			#WhyWeTweetMH - to attempt to battle stigma; to share information or signpost helpful resources	0	0
			#WhyWeTweetMH - to give people the chance to take part in research when they might not usually hear about opportunities via more traditional avenues	0	0
37	United Kingdom	Personal experience	To raise awareness, fight stigma & show myself and other people that we're not on our own #WhyWeTweetMH	1	2
38	United Kingdom	Personal experience	I tweet about my work, life & God. To not tweet about mental health would be excluding crucial elements of my relationship with all three! #WhyWeTweetMH	0	4
39	United States	Unknown	To inform, empower and inspire. We must advocate for and show others how to advocate for themselves. #WhyWeTweetMH	3	1
40	United Kingdom	Unknown	To raise awareness of the knock-on-effect of PTSD for families of the UK forces #WhyWeTweetMH	8	3
41	United Kingdom	Unknown	We do not only need to challenge stigma we have to eliminate stigma! Stigma is a social construct and so it is in our gift to	12	9

			influence #WhyWeTweetMH		
			Suicide might be complicated but extending a hand to someone is simple and it may save their life #ConnectingwithTwitter and share hope #WhyWeTweetMH #spsm	11	5
42	United Kingdom	Personal experience	I tweet about my daily life #WhyWeTweetMH	0	2
43	United Kingdom	Personal experience	If it helps even one person recognise they aren't alone in their pain #WhyWeTweetMH	3	0
44	United Kingdom	Unknown	#WhyWeTweetMH to give hope of recovery, confront stigma, influence improvement in services.	1	1
45	United Kingdom	Personal experience	#WhyWeTweetMH support & awareness is vital. I was previously given a diagnosis of M.A.D.D, which is not a constructive term. #Menhavetotalkmore	0	0
			#WhyWeTweetMH there are too many people who view it as a flaw and punish themselves or other people. #PrejudiceIsAnIssue	0	1
			#WhyWeTweetMH Strongly believe in being upfront about it because it's normal and is similar to lots of other illnesses, comes in lots of forms	0	0
46	Canada	Unknown	#WhyWeTweetMH to obliterate stigma and provide people with hope	1	1
47	United States	Professional experience	#WhyWeTweetMH share resources with professionals and advocates for suicide prevention, young people and healthcare integration	7	3
48	United Kingdom	Unknown	#WhyWeTweetMH ignorance & silence lets stigma grow and we can do so much more as a community	3	1
49	South Africa	Unknown	#WhyWeTweetMH to show people that they're not on their own and #mentalhealth will be #everyonesbusiness	1	1
50	Australia	Unknown	#WhyWeTweetMH vast range of resources & numerous sources. Mental health - we need to talk	1	0
51	United States	Personal experience	#WhyWeTweetMH An advocate told me that my voice was required on here to confront the 'Master Narratives' about mental health, trauma and suicide	3	5
52	United States	Personal experience	#WhyWeTweetMH because you cannot tell just by looking at me and by tweeting about it perhaps another person will know that they are not on their own	3	1
			Because I want my school, doctors, community and society to realise that they will not be able to dismiss us #WhyWeTweetMH	0	0
53	Unknown	Unknown	#WhyWeTweetMH To share, learn and flourish for patients, friends and families. Talking destroys stigma	0	0
54	United States	Personal experience	Because seeing another person is feeling the same helps me. #WhyWeTweetMH	0	0
55	Unknown	Professional experience	#WhyWeTweetMH To connect people and share resources, ideas and research that improve care for people with mental health disorders	0	1

56	United Kingdom	Unknown	Interesting concept #WhyWeTweetMH - emerging platform facilitating unique construction of shared understanding in mental health problems	0	0
57	Unknown	Personal experience	#WhyWeTweetMH sharing collective experiences is the reason I tweet. Mental illness regrettably thrives on isolation #Community	0	2
58	United States	Personal experience	To share my experiences & help other people	0	0
59	United States	Unknown	to increase awareness, to inform, to lessen stigma, to show that we care! #WhyWeTweetMH	1	2
60	United States	Unknown	It's time to tell people that you can have a mental illness and still have a productive life #endstigma #WhyWeTweetMH	0	0
61	United States	Unknown	Completely! Twitter is also fantastic for creating mental health communities & meeting people! :) #WhyWeTweetMH	1	0
62	United States	Unknown	I tweet about suicide prevention and mental health because it effects everybody, but it is underfunded in the United States #WhyWeTweetMH	0	0
63	United States	Personal experience	I tweet & blog about mental health to combat stigma and tell my story to (hopefully) inspire other people #WhyWeTweetMH	3	4
64	United States	Unknown	#susocialwork Build awareness!!	0	1
65	Unknown	Personal experience	I tweet humour to show people that there is light at the end of the dark tunnel. And I try my hardest to #EndTheStigma #WhyWeTweetMH	1	1
66	Unknown	Unknown	Brilliantly summed up! #Recognise #Reeducate #Reduce #Drinkwiseagewell	0	1
67	United States	Professional experience	#WhyWeTweetMH: Because we want teenagers to understand that there are non-judgmental, supportive services that are available	0	1
68	United States	Professional experience	Because I work with people with mental health diagnosis & appreciate its impact #SUSocialWork #WhyWeTweetMH	1	1
69	United States	Unknown	For resources! #SuicidePrevention #HealthcareIntegration	4	3
70	Unknown	Unknown	To raise awareness, stop stigma, create networks & build empathy & compassion is #WhyWeTweetMH	0	1
71	Unknown	Unknown	#WhyWeTweetMH So that people know that they are not on their own #ShareInspireConquer	0	0
			#WhyWeTweetMH to help people see that #suicide is not the answer	0	0
72	Unknown	Personal experience	It is a method to support myself & other people who experience the same, in an attempt to overcome how we are seen #WhyWeTweetMH	0	0
73	United Kingdom	Personal experience	Because it is too easy to place us in boxes; it is an element of me, but it doesn't define who I am #WhyWeTweetMH	1	0
74	United	Personal	#WhyWeTweetMH to remember that I am not the only person	0	1

	Kingdom	experience	who feels this way, to help people think differently about what people with		
			mental health problems look like, to assist others in finding information about mental health concerns, to raise awareness of #cyclothymia, in particular. #WhyWeTweetMH	1	2
75	United Kingdom	Personal and professional experience	#WhyWeTweetMH Because I've got 1st & 2nd hand experience & I also work in mental health. More empathy is needed	1	1
76	United Kingdom	Unknown	#WhyWeTweetMH To attempt to fight stigma and demonstrate to others battling mental health concerns that they are not on their own	1	0
77	United Kingdom	Professional experience	It is my job, but it is additionally a passion. On Twitter I can blur boundaries & keep it real. Twitter also saves lives. #WhyWeTweetMH	2	3
			Isolation and loneliness follow people who are set aside to live in poverty & with no hope. Twitter can connect those who are disconnected #WhyWeTweetMH	5	3
78	Unknown	Unknown	#WhyWeTweetMH Because #MentalHealthWeek2015 should not end after only 1 week. It is permanently time to talk about mental health.	12	8
			Because we have to stop stigma & begin speaking about what's actually important #WhyWeTweetMH	7	1
			Because my children, all children, are entitled to a better health care system for the future. #WhyWeTweetMH	11	1
79	Unknown	Unknown	#WhyWeTweetMH Because I might not know the storms my friends that have it experience but I am happy to offer my heart	0	2
80	United Kingdom	Unknown	#WhyWeTweetMH to eliminate the stigma that still impacts people in 2015	1	0
81	United Kingdom	Personal experience	To share my experiences with others and communicate with an amazing support network which is crucial in my continuing recovery #WhyWeTweetMH	2	1
82	United Kingdom	Professional experience	Discuss your experiences with mental health services to aid improvements in the care provided for others #WhyWeTweetMH	5	1
83	United Kingdom	Professional experience	To lessen stigma, circulate research & speak with people about their experiences of mental health #WhyWeTweetMH	0	0
84	United Kingdom	Unknown	For myself, it is to circulate info. & thoughts with people who care. Why do you tweet? #WhyWeTweetMH	1	1
85	United Kingdom	Personal experience	#WhyWeTweetMH Twitter is a way to escape from the real world. You are able to communicate with lots of people who you don’t know	0	0
86	United Kingdom	Unknown	#WhyWeTweetMH Tweeting about mental health helps people obtain helpful info. that they would not normally hear about	0	0
87	United Kingdom	Personal experience	I have been bereaved by suicide and have tried #WhyWeTweetMH - to campaign, learn, educate,	0	1

			communicate with people with similar experiences and beyond		
88	United Kingdom	Personal experience	The reasons why people tweet about mental health are: 1. to increase awareness, 2. to educate, 3. to share their experiences. #WhyWeTweetMH	1	0
89	Unknown	Unknown	#WhyWeTweetMH #MHawareness	0	0
90	Unknown	Unknown	To being buried, misjudged and shameful disorders out of the darkness. To relieve the struggle of those still to come. #WhyWeTweetMH	5	2
